# The Role of Deep Neuromuscular Blockade and Sugammadex in Laparoscopic Hysterectomy: A Randomized Controlled Trial

**DOI:** 10.3390/jcm14176163

**Published:** 2025-08-31

**Authors:** Corrado Terranova, Lorenzo Schiavoni, Francesco Plotti, Fabio Costa, Laura Feole, Stefania Rampello, Fernando Ficarola, Roberto Montera, Federica Guzzo, Daniela Luvero, Violante Di Donato, Alessia Mattei, Roberto Angioli, Carlo De Cicco Nardone

**Affiliations:** 1 Department of Gynecology, Fondazione Policlinico Universitario Campus Bio-Medico, Via Alvaro del Portillo, 200, 00128 Roma, Italy; 2 Research Unit of Gynecology Oncology, Department of Medicine and Surgery, Università Campus Bio-Medico di Roma, Via Alvaro del Portillo, 21, 00128 Roma, Italy; 3 Department of Anesthesia and Intensive Care, Fondazione Policlinico Universitario Campus Bio-Medico, 00128 Rome, Italy; 4 Obstetrics and Gynecological Unit, Department of Woman’s and Child’s Health, San Camillo-Forlanini Hospital, 00152 Rome, Italy; 5 Department of Gynecological, Obstetrical and Urological Sciences, “Sapienza” University of Rome, 00185 Rome, Italy

**Keywords:** postoperative pain, PONV, gynecology, neuromuscular recovery, neuromuscular blockade, laparoscopic hysterectomy

## Abstract

**Background/Objectives**: Laparoscopic gynecologic surgery is widely utilized due to its minimally invasive nature. Postoperative discomfort, including intra-abdominal and referred shoulder pain, remains a challenge. This study evaluates the impact of deep neuromuscular blockade (NMB) reversed with sugammadex compared to moderate NMB reversed with neostigmine on postoperative pain, recovery, and surgical conditions in patients undergoing laparoscopic hysterectomy. **Methods**: This double-blind, randomized controlled trial included 228 patients undergoing laparoscopic hysterectomy under standardized pneumoperitoneum pressure (12 mmHg). Participants were randomized into two groups: deep NMB with sugammadex (SUG) and moderate NMB with neostigmine (NEO). Primary outcomes included postoperative pain (NRS) and neuromuscular recovery time (TOF ratio ≥ 0.9). Secondary outcomes were surgical conditions, surgeon satisfaction, extubation and recovery times, incidence of postoperative nausea and vomiting (PONV), and analgesic consumption. **Results**: The SUG group exhibited lower pain scores up to 24 h compared to the NEO group (*p* < 0.05). Pain reductions remained statistically significant up to 6 h postoperatively after Bonferroni correction, while differences beyond this time were not significant after adjustment. Neuromuscular recovery was markedly faster in the SUG group (147.58 ± 82.26 sec vs. 488.02 ± 223.07 sec, *p* < 0.05). Patients in the SUG group had shorter extubation (ΔT1), awakening (ΔT2), and recovery room transfer times (ΔT3). PONV was significantly lower in the SUG group. Deep NMB did not contribute to the improvement of surgical workspace conditions. **Conclusions**: Deep NMB with sugammadex enhances postoperative pain control and accelerates neuromuscular recovery in laparoscopic hysterectomy. These findings support the adoption of deep NMB with sugammadex as a valid anesthetic approach in laparoscopic hysterectomy procedures.

## 1. Introduction

Laparoscopic surgery is one of the most performed procedures in gynecological practice due to its minimally invasive nature. It offers advantages such as smaller incisions, reduced postoperative pain, and shorter hospital stays compared to traditional laparotomy [1]. The creation of a surgical workspace in laparoscopy requires the insufflation of carbon dioxide (CO_2_) into the abdominal cavity, with intra-abdominal pressure (IAP) typically maintained at 12–15 mmHg (standard pressure) or reduced to 6–10 mmHg in low-pressure pneumoperitoneum [2,3,4].

Despite its benefits, up to 70% of gynecological patients experience postoperative pain. This discomfort originates from three primary components: deep intra-abdominal pain, superficial or incisional pain, and referred shoulder pain [3,4]. Pneumoperitoneum is believed to be the leading cause of shoulder pain, as CO_2_ insufflation results in diaphragmatic and phrenic nerve irritation and stretching. Additional contributing factors include residual intra-abdominal CO_2_, hemoperitoneum, and intraperitoneal acidosis [5,6].

Several studies have shown that low-pressure pneumoperitoneum reduces postoperative pain compared to standard-pressure pneumoperitoneum [3,7]. However, Madsen et al. found that the combination of low-pressure pneumoperitoneum and deep neuromuscular blockade (NMB) significantly reduced postoperative pain incidence following laparoscopic hysterectomy [6]. Nevertheless, it remains unclear whether this benefit arises from the lower IAP itself or the deep NMB [8,9]. A randomized trial by Park et al. suggested that deep NMB improves surgical conditions and may also have oncologic benefits by enhancing lymph node retrieval [9].

Emerging evidence indicates that deep NMB may improve surgical conditions by enhancing abdominal wall compliance, reducing intraoperative muscle contractions, and optimizing the surgical workspace. Additionally, it has been associated with reduced postoperative pain scores in bariatric surgery [5,6,10,11].

This double-blind, randomized controlled trial aims to evaluate the effect of deep NMB reversed with sugammadex compared to moderate NMB reversed with neostigmine on postoperative pain in patients undergoing laparoscopic hysterectomy, maintaining a standardized pneumoperitoneum pressure of 12 mmHg. Additionally, this study seeks to confirm the superiority of sugammadex over neostigmine in facilitating faster recovery from NMB. The findings of this research may contribute to optimizing anesthetic protocols and improving postoperative outcomes in gynecologic laparoscopy.

## 2. Materials and Methods

This randomized, controlled, double-blind trial was conducted by the Department of Gynecology at Campus Bio-Medico of Rome from April 2018 to December 2024 (No. NCT03519633; https://clinicaltrials.gov/ct2/show/NCT03519633, accessed on 26 April 2018), following the CONSORT (CONsolidated Standards of Reporting Trials) guidelines. The study adhered to the regulatory standards of Good Clinical Practice and the Declaration of Helsinki (1996) and was approved by the Internal Review Board of Campus Bio-Medico of Rome (No. 39/17 INT ComEtCBM, date 12 July 2017). Written informed consent was obtained from each patient included in the study.

We included women scheduled to undergo laparoscopic subtotal or total hysterectomy under general anesthesia requiring tracheal intubation.

Inclusion criteria: Age between 18 and 75 years, BMI between 16 and 40 kg/m^2^, ECOG Performance Status 0–1, American Society of Anesthesiologists (ASA) classification 1–3, and ability to provide informed consent.

Exclusion criteria: Pregnancy; active or recent pelvic inflammation; anticipated airway difficulty; history of allergy to rocuronium, neostigmine, or sugammadex; allergy to NSAIDs (Non-Steroidal Anti-Inflammatory Drugs); previous opioid use for chronic pain; patients receiving medications that may alter rocuronium’s duration of action (e.g., aminoglycosides and magnesium); hepatic or renal failure; persistent coagulopathy; neurological or cognitive disorders; conversion to laparotomy; and onset of intraoperative complications.

**Protocol Compliance**

No protocol deviations occurred during the study. All randomized patients received the allocated intervention and were included in the final analysis (intention-to-treat population).

Patients were randomly assigned to either deep NMB with sugammadex for reversal (SUG group) or moderate NMB with neostigmine plus atropine for reversal (NEO group). Randomization was performed using a computer-generated random number of series via Microsoft^®^ Excel 2016 (version 16.0). Patients were randomized using a computer-generated sequence in blocks of six with a 1:1 allocation ratio. Stratification was not performed. Allocation concealment was ensured using sealed, opaque envelopes prepared and opened by an independent staff member not involved in patient care or data collection. Group allocation was recorded on an Excel sheet, stored in an opaque envelope, and placed in a locked drawer accessible only to the investigator. Anesthesiologists were aware of the treatment assignment, while the surgeon and surgical staff were blinded. Patients and postoperative care providers, including nurses and physicians involved in pain assessment and analgesic administration, were blinded to treatment allocation, and the statistician was blinded. Postoperative pain scores were recorded by trained nurses blinded to treatment allocation, using a standardized numerical rating scale (NRS), minimizing inter-observer variability.

Primary outcomes included postoperative pain within the first 48 h after surgery and reversal time from NMB (train-of-four (TOF) ratio ≥ 0.9). Secondary outcomes included surgical conditions, surgeon satisfaction, anesthesia duration, operation time, extubation time, awakening time, operating room discharge time, incidence of dry mouth and postoperative nausea and vomiting (PONV), need for rescue analgesics (paracetamol, ketorolac and morphine) and antiemetics (metoclopramide), and resolution of postoperative ileus.

Anesthesia was managed with intraoperative monitoring, including a three-lead electrocardiogram, blood pressure, heart rate, pulse oximetry, bispectral index monitoring, and intra-abdominal pressure (IAP). Neuromuscular function was monitored using acceleromyography, measuring the adductor pollicis muscle response via a thumb-placed sensor. The TOF was assessed, and if no thumb twitches were detected, the post-tetanic count (PTC) was measured.

Anesthesia induction included fentanyl (3 mcg/kg), propofol (2–2.5 mg/kg), and rocuronium (0.6 mg/kg for the NEO group, or 1 mg/kg for the SUG group). Before rocuronium administration, the acceleromyography device was calibrated to obtain a baseline TOF ratio. Tracheal intubation was performed two minutes after rocuronium administration. Anesthesia maintenance included desflurane (6%) and remifentanil TCI (2–4 ng/mL) as required. Rocuronium was continuously infused and adjusted to maintain TOF response at 1–2 in the NEO group, or PTC at 1–2 in the SUG group. Preventive analgesia included paracetamol (1 g), dexamethasone (0.1 mg/kg), clonidine (2 mcg/kg), and granisetron (1 mg).

After intubation, patients were placed in the lithotomy position, and hemodynamic stability was assessed before surgery. During laparoscopy, patients were positioned in Trendelenburg to facilitate bowel displacement from the pelvis. Surgical conditions and surgeon satisfaction were evaluated by the same lead surgeon throughout the study every 20 min, using a five-point Surgical Rating Scale (SRS) adapted from the literature [8,10,12], ranging from 1 (extremely poor) to 5 (optimal). Surgeon satisfaction was assessed regarding neck strain, back strain, visual acuity, and overall satisfaction using a five-point scale (1 = very dissatisfied to 5 = very satisfied).

At the end of surgery, hemostasis was achieved, CO_2_ was evacuated, and trocars were removed. The patient was repositioned horizontally, and incisions were sutured. NMB reversal was initiated during hemostasis using either sugammadex (SUG group) or neostigmine plus atropine (NEO group), titrated according to TOF results. Once TOF ≥ 0.9 was achieved, desflurane was discontinued, the patient was awakened, and extubation was performed.

Postoperative analgesia began 20 min before surgery ended, including ketorolac (90 mg i.v. for the first 24 h). PONV prophylaxis consisted of granisetron (3 mg i.v. for 24 h) and ranitidine (50 mg i.v. daily for 48 h). In the post-anesthesia care unit (PACU), blinded personnel recorded PONV incidence and rescue analgesic/antiemetic use. Pain levels were recorded for the first 48 h at multiple time points (30 min, 60 min, 120 min, 6 h, 12 h, 24 h, 36 h, and 48 h), using a 0–10 numerical rating scale (NRS). Rescue medications included paracetamol (1 g, NRS > 4 or T > 38 °C, max 3 g/day), ketorolac (30 mg, after 24 h, NRS > 6, max 90 mg/day), morphine (2 mg/hourly, NRS > 8, max 6 mg/day), and metoclopramide (10 mg i.v. for nausea).

The trial was supervised by a senior anesthesiologist and a clinical research coordinator, who monitored adherence to the study protocol and adverse-event reporting.

Sample size calculation and statistical analysis: Based on Barron’s study, which reported a reduction in pain from 4.07 to 2.79 (with a common standard deviation of 2.3), the required sample size was 107 patients per group (α = 0.01; β = 0.10) [13]. Statistical differences between groups were tested using Student’s t-test for normally distributed data or the Mann–Whitney test for non-normally distributed data. Nominal data were presented as absolute numbers with percentages and compared using the χ^2^ test or Fisher’s exact test. A *p*-value < 0.05 was considered statistically significant. Analyses were performed using SigmaPlot^®^ software version 14.0. Post hoc power analyses were performed for key secondary outcomes based on the observed effect sizes (Cohen’s d and h). The achieved power was ≥ 0.84 for all tested comparisons, including analgesic consumption, PONV incidence, and extubation time, confirming the adequacy of the sample size in detecting clinically relevant between-group differences across both primary and secondary endpoints. Effect sizes were calculated to estimate the magnitude of between-group differences. Cohen’s d was used for continuous variables, and Cohen’s h for binary outcomes. Given the multiple pain assessments at different time points and conditions, a Bonferroni correction was applied to account for multiplicity.

## 3. Results

**Figure 1:**
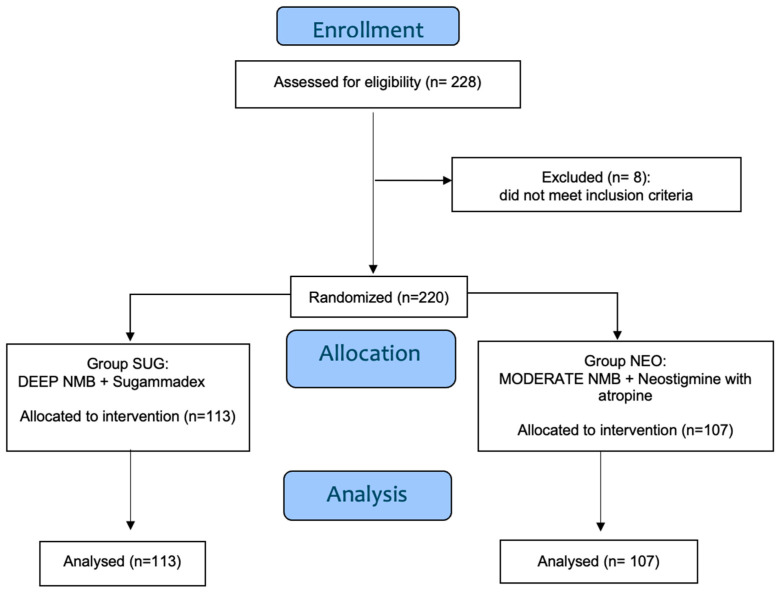
Study flowchart (CONSORT Flow Diagram).

We initially enrolled in this study 228 women scheduled for laparoscopic hysterectomy under general anesthesia, requiring tracheal intubation. Eight patients were excluded before randomization due to meeting one or more exclusion criteria. In total, 220 patients were included in the final analysis, with 113 assigned to the SUG group and 107 to the NEO group. Figure 1 shows the flowchart of the study. Table 1 presents patients’ characteristics, demonstrating no significant differences between the two groups in terms of age and preoperative measures.

Postoperative measures of Hb, WBC, PCR, and body temperature were similar in the first two days following surgery.

Intraoperative data (Table 2) showed no significant differences between the groups in terms of total CO_2_ insufflated, insufflation flow, and insufflation time. However, we found that reversal time was significantly lower in the SUG group (147.58 ± 82.26 vs. 488.02 ± 223.07; 95% CI: 132.3–162.9 vs. 445.3–530.8), with a large effect size (Cohen’s d = –2.05).

There were no significant differences in anesthesia and surgery times between the two groups, but extubation time (ΔT1), awakening time (ΔT2), and transfer to the recovery room (ΔT3) were significantly shorter in the SUG group (ΔT1: 7.98 ± 3.87 vs. 13.40 ± 8.27, 95% CI: 7.26–8.70 vs. 11.81–14.99, d = –0.85; ΔT2: 7.51 ± 3.75 vs. 12.73 ± 7.81, 95% CI: 6.81–8.21 vs. 11.23–14.23, d = –0.86; ΔT3: 12.37 ± 5.21 vs. 17.78 ± 7.74, 95% CI: 11.40–13.34 vs. 16.30–19.26, d = –0.82).

Finally, postoperative ileus resolution occurred faster in the SUG group (14.03 ± 6.3 h vs. 19.56 ± 8.2 h, 95% CI: 12.86–15.20 vs. 17.99–21.13), with a moderate-to-large effect size (d = –0.76) (Table 2).

Pain scores, measured using the NRS scale, are detailed in Table 3. The SUG group reported significant lower pain at each time point until 6 h (30 min, 1 h, 2 h, and 6 h). The between-group difference in pain at 30 min at rest showed a large effect size (Cohen’s d = –1.30). Across the other conditions (rest, palpation, movement, and cough), pain was significantly lower in the SUG group until 24 h. Data regarding pain after 24 h did not significantly differ between the two groups. After applying Bonferroni correction for 24 comparisons (adjusted α = 0.0021), the between-group differences in postoperative pain scores remained statistically significant across all conditions (rest, palpation, movement, and cough) up to 6 h after surgery (all *p* < 0.001). In contrast, differences observed at 12 and 24 h (uncorrected *p*-values ranging from 0.01 to 0.04) did not retain statistical significance after correction and should be considered exploratory.

The second primary outcome, the time to reversal of NMB (TOF ratio ≥0.9), was significantly shorter in the SUG group compared to the NEO group (147.58 ± 82.26 vs. 488.02 ± 223.07, 95% CI: 132.3–162.9 vs. 445.3–530.8, *p* < 0.05).

Surgeon satisfaction was not significantly influenced in our study. Intraoperative surgeon satisfaction assessed through the SRS (1–5 scale) showed similar scores in the SUG group compared to the NEO group at all time points (i.e., overall satisfaction, 60 min (4.6 ± 0.6 vs. 3.8 ± 0.8, Cohen’s d = 1.14)).

Analgesic consumption during PACU stay was significantly lower in the SUG group (16%, 95% CI: 9.2–22.7) compared to the NEO group (38%, 95% CI: 29.1–47.5, *p* < 0.05, Cohen’s h = –0.51). In addition, rescue analgesic use during the first 48 h was reduced in the SUG group, with significantly less morphine used on day 0 (2%, 95% CI: –0.7–4.2 in SUG vs. 13%, 95% CI: 6.7–19.5 in NEO; *p* < 0.05, Cohen’s h = –0.45) and less ketorolac on day 1 (27%, 95% CI: 19.2–35.7 in SUG vs. 46%, 95% CI: 36.4–55.2 in NEO; *p* < 0.05, Cohen’s h = –0.40). Interestingly, the overall use of analgesics was 119 doses in the SUG group and 178 doses in the NEO group (*p* < 0.001).

PONV incidences were significantly lower in the SUG group during PACU (21%, 95% CI: 13.7–28.8 vs. 49%, 95% CI: 39.1–58.1, *p* < 0.05, Cohen’s h = –0.60) and remained lower until 6 h post-surgery (e.g., 30 min: SUG 19%, 95% CI: 11.4–25.8 vs. NEO 57%, 95% CI: 47.6–66.4, *p* < 0.05, Cohen’s h = –0.81).

Correlation analyses between postoperative pain, PONV, recovery time, and analgesic consumption did not reveal any statistically significant associations. Pearson and Spearman correlation coefficients ranged from –0.11 to 0.09, with all *p*-values > 0.69, suggesting no meaningful linear or monotonic relationships among these variables in our cohort.

The length of hospital stay did not differ significantly between the two groups.

No cases of hypersensitivity or anaphylaxis related to sugammadex were observed in our study. No intraoperative complications, including bleeding, visceral injury, or need for conversion to laparotomy, occurred in either group. Additionally, no adverse events related to deep NMB were reported.

## 4. Discussion

Effective pain management is essential to enhance patient recovery following laparoscopic procedures. Evidence suggests that deep NMB may provide significant advantages over moderate NMB. Our study contributes to this growing body of knowledge by demonstrating that deep NMB, reversed with sugammadex, is associated with reduced postoperative pain, faster recovery, and a lower incidence of PONV compared to moderate NMB reversed with neostigmine.

Our findings align only in part with the current literature. While some randomized trials and meta-analyses [5,13,14] report improved surgical conditions and faster recovery with deep NMB, others show minimal clinical benefit, particularly when standard-pressure pneumoperitoneum is used. These discrepancies may reflect methodological heterogeneity, including variation in NMB depth monitoring, reversal agents, surgical type, and pressure settings. On the other hand, our findings are consistent with recent data by Kathopoulis et al. [15], who similarly demonstrated improved pain control and recovery parameters in patients receiving deep NMB during gynecologic laparoscopic procedures. This alignment reinforces the clinical relevance of our results.

Postoperative pain following laparoscopic surgery originates from multiple sources, including intra-abdominal pain, superficial incisional pain, and referred shoulder pain mainly due to diaphragmatic irritation from CO_2_ insufflation [4,14]. While postoperative pain after open surgery is mostly of somatic origin, postoperative pain after laparoscopic surgery consists of both somatic and visceral elements. The insertion of trocars and sutures is responsible of somatic pain, which is referred to as abdominal pain. In contrast, visceral referred pain after laparoscopy is described as moderate-to-severe dull pain in the shoulder, scapula, and abdomen. Peritoneal stretching, inflammation, postoperative gas retention, and muscle spasm mainly cause this type of pain. Some of these mechanisms can be attenuated, for example, by correct surgical manipulation, optimizing the temperature and humidity of the gas, and decreasing the insufflation pressure. As regards the pathophysiology of pain related to muscle stretching, it may be further explained by a recent study in a rat model [12], which demonstrated that muscle contraction significantly activated nociceptive dorsal horn neurons (DHN). When NMB was induced with pancuronium, muscle contractions and DHN activation were inhibited, confirming that contraction-induced activation was in part responsible for pain. These findings suggest a potential analgesic effect of deep NMB, as it reduces muscle stretching, which triggers pain stimuli.

Our study showed that patients receiving deep NMB (SUG) reported lower pain scores within the first 6 h compared to those in the moderate NMB group (NEO), after Bonferroni correction for multiple comparisons. The mean pain scores were consistently lower in the SUG group across different conditions (rest, palpation, movement, and cough), suggesting a tangible benefit of deep NMB in minimizing postoperative discomfort. Although pain scores showed statistically significant differences between groups, the magnitude of reduction (2–3 points on the NRS) during the early postoperative period is also considered clinically meaningful in the surgical setting. More specifically, the observed reductions in pain scores (2–3 NRS points) exceed the Minimal Clinically Important Difference (MCID) for postoperative pain, which has been estimated at approximately 1.3–1.5 points on the NRS in surgical populations [16,17]. Therefore, these findings not only reached statistical significance but also fulfilled the accepted threshold for clinical relevance, reinforcing their impact for patient recovery and analgesic management.

Importantly, the analgesic benefit of deep neuromuscular blockade with sugammadex was confirmed in the early postoperative period, where differences remained significant after Bonferroni correction for multiple comparisons. Beyond 6 h, the observed reductions in pain scores did not retain statistical significance once corrected for multiplicity, although they showed a consistent trend in favor of sugammadex. These findings suggest that the most robust advantage of this anesthetic strategy is concentrated in the immediate postoperative phase, while later differences should be interpreted with caution as exploratory results.

The observed benefits in recovery and analgesic sparing in the SUG group may also be explained by more rapid and complete reversal of NMB, reducing residual paralysis and facilitating earlier extubation. Additionally, sugammadex avoids the cholinergic effects of neostigmine, which may contribute to reduced incidence of PONV and ileus. Enhanced postoperative bowel function may also relate to lower opioid requirements in the immediate postoperative period.

These findings align with prior research, such as Madsen et al.’s work, which demonstrated that deep NMB and low-pressure pneumoperitoneum together reduce postoperative pain after laparoscopic hysterectomy [5]. Unlike Madsen’s study, our trial maintained a standardized pneumoperitoneum pressure of 12 mmHg in both groups, confirming that the observed pain reduction was attributable to the NMB depth rather than differences in intra-abdominal pressure. Reduced intra-abdominal pressure alone is, however, correlated with less postoperative pain, as demonstrated in numerous studies [15,18,19]: the physio-pathological causes, as previously stated, are multiple, but an important role is played by the different degree of muscular inflammation correlated to a different tension of the abdominal muscles.

Kathopoulis et al. demonstrated that deep NMB was associated with lower pain scores and reduced analgesic consumption in gynecologic laparoscopic procedures [15]. In his study, Kathopoulis included patients receiving laparoscopy for various gynecologic procedures, while we included only patients undergoing laparoscopic hysterectomy to have a more homogeneous population of study. Moreover, in our study, analgesic consumption was significantly lower in the SUG group, particularly regarding opioid use. Morphine administration was required in only 2% of patients in the SUG group compared to 13% in the NEO group on day 0, and ketorolac use on day 1 was also significantly lower. These findings are consistent with those reported by Castro et al., who observed that patients reversed with sugammadex after bariatric surgery experienced less postoperative pain and lower PONV rates compared to those reversed with neostigmine [10].

While improved surgical conditions were anticipated with deep NMB, our findings indicate that surgeon satisfaction, measured by the Surgical Rating Scale (SRS), did not differ significantly between the two groups, as recently demonstrated by Kathopoulis et al. [15]. This result contrasts with previous studies that reported better surgical working conditions using deep NMB; for example, Dubois et al. [4] demonstrated superior surgical field in patients under deep NMB. Similarly, Raval et al. observed enhanced surgical visualization and marginally shorter operative times with deep NMB during abdominal procedures [13]. Additionally, Esa et al. found improved surgical workspace conditions with deep NMB in the context of low-pressure pneumoperitoneum, emphasizing its potential benefit in such settings [19].

A possible explanation for this discrepancy lies in the different pneumoperitoneum pressures adopted across studies. In most of the above-mentioned trials, surgeries were performed under low intra-abdominal pressures (typically 6–10 mmHg), where the impact of deep muscle relaxation on abdominal wall compliance and workspace expansion is more pronounced. In contrast, our study was conducted under a constant and standard pneumoperitoneum pressure of 12 mmHg, which is already sufficient to provide an adequate surgical field. At this pressure level, the incremental benefit of deep NMB on surgical visibility might be minimal or clinically irrelevant, thus explaining the lack of difference in surgeon satisfaction in our findings. Therefore, we believe that the effect of deep NMB on surgical field conditions is likely pressure-dependent and becomes more evident only in low-pressure settings.

A key finding of our study was the significantly shorter neuromuscular recovery time in the deep NMB group. The time to achieve a TOF ratio of ≥ 0.9 was markedly reduced in the SUG group (147.58 ± 82.26 sec) compared to the NEO group (488.02 ± 223.07 sec, *p* < 0.05). This rapid recovery facilitated faster emergence from anesthesia, as evidenced by a shorter extubation time (∆T1), awakening time (∆T2), and transfer to the recovery room (∆T3) in the SUG group. Similar results were observed by Putz et al., who reported that patients receiving deep NMB reversed with sugammadex experienced significantly faster operating room discharge compared to those reversed with neostigmine (22 min vs. 72 min, *p* < 0.05) [20].

The incidence of PONV was significantly lower in the SUG group, both in the PACU and throughout the 48 h postoperative period. This finding aligns with previous research by Ledowski et al., who found that sugammadex use was associated with lower PONV rates [21]. Similar findings were reported by Yagan et al., who observed a lower incidence of PONV in patients reversed with sugammadex compared to neostigmine [22].

Effect sizes for categorical outcomes were calculated using Cohen’s h and ranged from small to large. Notably, moderate-to-large effects were observed for analgesic use and PONV reduction, supporting the clinical relevance of the findings beyond statistical significance.

Although sugammadex has been associated with rare but potentially severe adverse events—particularly in older and pediatric populations—it remains a widely used and generally well-tolerated agent. Clinicians should be aware of these potential reactions to ensure prompt and appropriate management when necessary [23]. On the other hand, large-scale studies such as that by Reutzler et al. have reported similar safety profiles between sugammadex and neostigmine, with only a small proportion of patients experiencing postoperative side effects [24]. In our cohort, no sugammadex-related adverse events or complications associated with deep NMB were observed, supporting the safety of this strategy in routine gynecologic laparoscopic procedures. Nonetheless, the possibility of rare hypersensitivity reactions should always be considered when assessing the overall risk–benefit profile of sugammadex.

While the duration of surgery itself remained unchanged between groups in our study, the reduced recovery time from anesthesia in the SUG group led to a decreased overall occupancy time in the operating room. In fact, the significantly faster neuromuscular recovery observed in the SUG group (mean difference > 5 min) is particularly relevant in busy surgical settings, where even modest time savings can contribute to improved perioperative workflow and reduced occupancy of operating or recovery rooms. Moreover, faster recovery may reduce the risk of residual paralysis and facilitate earlier patient mobilization and discharge from the PACU. We did not perform a cost-effectiveness analysis; further studies in the future may better analyze this important and interesting aspect.

Although our study provides robust evidence supporting the benefits of deep neuromuscular blockade (NMB), several limitations should be acknowledged. First, while our findings indicate clear advantages in ASA I–II patients undergoing laparoscopic hysterectomy, caution is warranted when extrapolating these results to other surgical populations, higher-risk patients (e.g., ASA III–IV), or procedures performed under low-pressure pneumoperitoneum. Further studies in these subgroups are needed to validate generalizability.

Additionally, recovery times were reported as means and standard deviations; however, future research could incorporate survival analysis techniques to more accurately evaluate time-dependent outcomes, such as reversal time or readiness for discharge.

Investigating deep NMB in procedures conducted exclusively under low pneumoperitoneum pressure could also offer insights, as reduced muscular stretching may further influence postoperative pain.

Lastly, a dedicated cost-effectiveness analysis comparing sugammadex and neostigmine would be valuable to better understand the economic implications of each strategy in clinical practice.

## 5. Conclusions

Our findings reinforce the clinical benefits of deep NMB in gynecologic laparoscopic surgery, demonstrating reduced postoperative pain, faster recovery, and lower PONV incidence compared to moderate NMB. The use of sugammadex for NMB reversal enhances these advantages by facilitating rapid recovery and minimizing residual curarization. Given these findings, deep NMB with sugammadex should be considered a component of anesthetic management for major laparoscopic gynecologic procedures, with potential implications for fast-track surgery protocols. Further research is warranted to confirm these benefits in larger and more heterogeneous patient populations to assess the long-term impact on healthcare efficiency.

## Figures and Tables

**Table 1:** Patient characteristics.

	SUG Group (n = 113)	NEO Group (n = 107)	*p*-Value
BMI (kg m^−2^)	24.2 ± 3.7	23.5 ± 4.3	0.39
Age (years)	44.8 ± 13.6	48.8 ± 10.0	0.06
ASA 1/2/3	19/37/1	10/40/2	0.18
Hb pre (g/dL)	13.1 ± 1.2	12.9 ± 1.1	0.41
WBC pre (× 10^9^/L)	6.9 ± 1.9	6.4 ± 1.5	0.11

**Table 2:** Intraoperative measurements. ns: not significant.

	SUG Group (*n* = 113)	NEO Group (*n* = 107)	*p*-Value
TOT CO_2_ insufflated (L)	436.6 ± 327.2	398 ± 302.4	0.52
CO_2_ insufflation flow (Lmin-1)	7.0 ± 4.5	6.8 ±3.7	0.81
CO_2_ insufflation time (min)	82.5 ±50.0	79.2 ±63.6	0.76
DURATION of anesthesia (min)	118 ± 56	114 ± 72	0.73
Reversal time: TOF ratio ≥0.9 (sec)	147.58 ± 82.26	488.02 ±223.07	< 0.05
Duration of surgery (min)	105.72 ± 57.43	101.65 ± 63.55	0.72
ΔT1 end surgery-extubation (min)	7.98 ± 3.87	13.40 ± 8.27	< 0.05
ΔT2 end surgery—awakening (min)	7.51 ± 3.75	12.73 ± 7.81	< 0.05
ΔT3 end surgery—recovery room (min)	12,37 ± 5,21	17,78 ± 7,74	< 0.05

**Table 3:** Postoperative pain analysis with NRS (1–10 scale). ns: not significant.

	NRS at 30 min, Postoperative				NRS at 1 h, Postoperative				NRS at 2 h, Postoperative			
	SUG (NRS ± SD)	NEO (NRS ± SD)	*p*-value	Significant after Bonferroni	SUG (NRS ± SD)	NEO (NRS ± SD)	*p*-value	Significant after Bonferroni	SUG (NRS ± SD)	NEO (NRS ± SD)	*p*-value	Significant after Bonferroni
Rest	3.8 ±2.5	6.6 ±1.7	<0.05	Yes	3.7 ± 2.3	6.7 ±1.6	<0.05	Yes	3.6 ± 1.9	6.5 ± 1.6	<0.05	Yes
Palpation	4.7 ± 2.6	7.5 ± 1.5	<0.05	Yes	4.7 ± 2.4	7.5 ±1.3	<0.05	Yes	4.7 ± 1.8	7.3 ± 1.1	<0.05	Yes
Movement	4.7 ± 2.5	7.6 ± 1.6	<0.05	Yes	4.6 ± 2.3	7.5 ±1.3	<0.05	Yes	4.6 ± 1.7	7.2 ± 1.1	<0.05	Yes
Cough	4.8 ± 2.6	7.6 ± 1.6	<0.05	Yes	4.7 ± 2.2	7.6 ±1.3	<0.05	Yes	4.7 ± 1.7	7.0 ± 1.1	<0.05	Yes
	NRS at 6 h, Postoperative				NRS at 12 h, Postoperative				NRS at 24 h, Postoperative			
	SUG (NRS ± SD)	NEO (NRS ± SD)	*p*-value	Significant after Bonferroni	SUG (NRS ± SD)	NEO (NRS ± SD)	*p*-value	Significant after Bonferroni	SUG (NRS ± SD)	NEO (NRS ± SD)	*p*-value	Significant after Bonferroni
Rest	3.3 ± 1.8	5.6 ± 1.6	<0.05	Yes	2.6 ± 1.8	2.9 ± 1.5	0.29	No	2.1 ± 1.7	2.7± 1.7	0.06	No
Palpation	4.4 ± 1.7	7.0 ± 1.2	<0.05	Yes	3.6 ± 1.9	4.5 ± 1.9	<0.05	No	2.8 ± 1.8	3.5 ± 1.8	<0.05	No
Movement	4.2 ± 1.7	7.0 ± 1.0	<0.05	Yes	3.6 ± 1.8	4.4 ± 2.1	<0.05	No	2.9 ± 1.7	3.7 ± 2.0	<0.05	No
Cough	4.4 ± 1.8	7.0 ± 1.1	<0.05	Yes	3.7 ± 1.8	4.6 ± 1.9	<0.05	No	3.1 ± 1.5	3.8 ± 2.3	<0.05	No

## Data Availability

The data presented in this study are available upon request from the corresponding author (data are not publicly available due to privacy).

## Abbreviations

The following abbreviations are used in this manuscript:

NMB	Neuromuscular blockade
PONV	Postoperative Nausea and Vomiting
IAP	Intra-abdominal pressure
CO_2_	Carbon dioxide
TOF	Train-of-four
PTC	Post tetanic count
BMI	Body mass index
PACU	Post-anesthesia care unit
SRS	Surgical Rating Scale
Hb	Hemoglobin
WBCs	White blood cells
PCR	C-reactive protein
